# Reduced Air Leakage During Non-Invasive Ventilation Using a Simple Anesthetic Mask With 3D-Printed Adaptor in an Anthropometric Based Pediatric Head–Lung Model

**DOI:** 10.3389/fped.2022.873426

**Published:** 2022-04-28

**Authors:** Renée Hovenier, Lyè Goto, Toon Huysmans, Monica van Gestel, Rozalinde Klein-Blommert, Dick Markhorst, Coen Dijkman, Reinout A. Bem

**Affiliations:** ^1^Pediatric Intensive Care Unit, Emma Children's Hospital, Amsterdam University Medical Centers, Location AMC, Amsterdam, Netherlands; ^2^Department of Technical Medicine, University of Twente, Enschede, Netherlands; ^3^Faculty of Industrial Design Engineering, Delft University of Technology, Delft, Netherlands; ^4^Imec-Vision Lab, Department of Physics, University of Antwerp, Antwerp, Belgium; ^5^Department for Medical Innovation and Development, Amsterdam University Medical Centers, Location AMC, Amsterdam, Netherlands

**Keywords:** children, non-invasive ventilation, interface, acute respiratory failure, 3D-printed, anthropometry

## Abstract

Non-invasive ventilation (NIV) is increasingly used in the support of acute respiratory failure in critically ill children admitted to the pediatric intensive care unit (PICU). One of the major challenges in pediatric NIV is finding an optimal fitting mask that limits air leakage, in particular for young children and those with specific facial features. Here, we describe the development of a pediatric head–lung model, based on 3D anthropometric data, to simulate pediatric NIV in a 1-year-old child, which can serve as a tool to investigate the effectiveness of NIV masks. Using this model, the primary aim of this study was to determine the extent of air leakage during NIV with our recently described simple anesthetic mask with a 3D-printed quick-release adaptor, as compared with a commercially available pediatric NIV mask. The simple anesthetic mask provided a better seal resulting in lower air leakage at various positive pressure levels as compared with the commercial mask. These data further support the use of the simple anesthetic mask as a reasonable alternative during pediatric NIV in the acute setting. Moreover, the pediatric head–lung model provides a promising tool to study the applicability and effectiveness of customized pediatric NIV masks in the future.

## Introduction

The use of non-invasive ventilation (NIV) in the pediatric intensive care unit (PICU) to provide respiratory support for children of all ages has increased substantially over the past three decades (1–5). NIV can be a good alternative for invasive mechanical ventilation, which necessitates an artificial airway, in selected pediatric patients, including those with acute, moderate (hypoxic) respiratory failure. Advantages of NIV include a lower risk of nosocomial infections and injury to the airways and lungs, and also a reduced need for sedation (6–8).

Although the use of NIV provides benefits, the effectiveness of this type of respiratory support is highly dependent on the fit of the interface, most commonly a face mask (6, 9–11). Currently, this is considered one of the most important challenges in NIV in children. Commercially available masks come in very limited shapes and sizes and do not appropriately address the large variation in the face shapes and sizes of children of different ages, or those with specific facial features in the context of a (genetic) syndrome (10, 12–17). As such, the pursuit of finding an optimal fitting NIV mask in daily clinical practice, particularly in young children younger than 4 years, can be highly demanding. The suboptimal fit of the NIV mask results in patient discomfort, patient-ventilator asynchrony, and serious air leaks, negating the supportive effect of positive pressure ventilation (6, 9, 18, 19). Moreover, painful pressure ulcers may arise even after short-term use (10, 15, 18, 19). Such complications contribute to the failure of NIV treatment, which occurs in up to 15–30% of pediatric cases in the PICU (5, 20).

Optimization of the interface fitting, including strategies that go so far as personalizing a mask by 3D scanning and printing techniques, holds a promising future to reduce air leakage, and hence, improve pediatric NIV effectiveness (16, 17, 21). Recently, we described the successful use of a simple anesthetic (nose–mouth) mask for NIV in a 4-year-old child with specific facial features admitted to the PICU for acute hypoxic respiratory failure (22). This anesthetic mask is connected to a 5-point headgear using an in-house developed, 3D-printed reusable quick-release adaptor, and may serve as an effective and relatively cheap alternative to commercially available NIV masks. In particular, anesthetic masks are available in several sizes, including for very young children, and thus, potentially provide a good fit in daily clinical practice.

Here, we describe the development of a 3D-printed pediatric head–lung model, based on age-related 3D anthropometric data, as a tool to further investigate the applicability and effectiveness of (customized) pediatric NIV masks in a controlled, simulated setting. In this study, we produced a pediatric head–lung model of a 1-year-old child, and aimed to determine the extent of air leakage at various positive pressure levels during NIV using the simple anesthetic mask, as compared to a commercially available NIV mask.

## Materials and Methods

### Pediatric Head–Lung Model

To create a setting for simulation of interface fitting and placement, and NIV in children, a 3D-printed pediatric head–lung model was developed. For the design of the model as used in this study, we obtained the 3D anthropometric data from the open-source database DINED (http://dined.io.tudelft.nl) (23), comprising a principal component analysis and regression model between age and the 3D head and face shape of 302 Dutch children (14), to create an enriched statistical shape model (SSM) of a 1-year old child (mean age: 12.3 ± 2.9 months) with the following dimensions: a sellion-promentale length of 66.8 ± 4.6 mm, a mouth width of 33.5 ± 4.0 mm, a nose width of 25.4 ± 1.3 mm, and a nose length of 27.5 ± 1.6 mm. The 3D-printed pediatric head–lung model consists of a hard and sturdy interior, surrounded by a soft silicone layer that mimics human skin.

The inner head was produced using in-house 3D printing with black acrylonitrile styrene acrylate (ASA) using the Fortus 450mc (Stratasys Ltd®, Valencia, CA, USA). For the design, the 3D (.STL) files of the enriched SSM were loaded into the computer-aided design (CAD) program Autodesk Inventor (Autodesk Inventor, Autodesk, San Rafael). The 3D-inner part was scaled down in all directions by the thickness of the desired outer silicone skin layer (5 mm) so that the final model with the outer layer would return to its exact original dimensions. To ensure an equal distribution of the outer silicone layer, several support protrusions with a corresponding height of 5 mm were added. In addition, the ears were flattened to provide a realistic soft feel, and an easier casting model. At last, a cavity was created inside the hard interior head model to mimic the upper airways (diameter 6 mm) with holes for the “nose” and “mouth,” connecting to a vertical hollow tube representing a “trachea,” which can be connected to a lung simulator, the adult/infant Test Lung model 560li (Michigan Instruments, Grand Rapids, MI, USA).

As a second step, a mold was designed for silicone casting. For this, the 3D head (.STL) file was subtracted from a solid box in Autodesk Inventor, leading to a negative of the head. This box was made as small as possible, and all dead space was removed to reduce printing time. The mold was subsequently split into several parts with connection points (using plugs and holes) in between. It was in-house printed with polycarbonate (PC)-ISO using the Fortus 450mc.

Finally, the outer mold and the inner head were assembled together in preparation for the creation of the skin layer using silicone casting. For this, EcoFlex 00-20 (Smooth on, Inc., Macungie, PA, USA), a soft-feeling and flexible silicone was used to mimic real human skin. Equal amounts of parts A and B were weighed and mixed in a cup. In addition, a few drops of medium flesh pigment (Neill's Materials, Suffolk, UK) were added to create a realistic skin color. The mixture was placed into a vacuum chamber for several minutes to allow any air inside to escape, thereby preventing air bubbles in the final head model. Hereafter, the silicone mixture was poured into the mold, between the inner head and outer mold. The mold was removed after hardening for approximately 4 h. A biopsy punch was used to make holes in the silicone to the hollow mouth and nose tubes to allow airflow through the model. Furthermore, talcum powder was applied over the silicone layer to reduce stickiness.

### Pediatric NIV Simulation

The pediatric head–lung model was used to simulate mask interface placement and NIV. In this study, two masks were compared: (1) a simple anesthetic mask (nose–mouth full face mask from Ambu®King, size 2, Denmark), and (2) a total face mask (Respironics PerforMax®, size XS, Phillips, The Netherlands) covering the mouth, nose, and eyes. The simple anesthetic mask was connected to a five-point headgear (Respireo Soft Child Non Vented Masks, Air Liquide Healthcare, Paris, France) by our in-house 3D-printed quick release adaptor as described previously (22). The total face mask was used with the four-point headgear as supplied by the manufacturer. In the range of sizes available for both masks, the aforementioned sizes were selected on the forehand of the study procedure based on what was deemed to provide the most optimal fitting.

During NIV simulation, the masks were connected to a mechanical ventilator Hamilton-C6 (Hamilton Medical®, Bonaduz, Switzerland) using Fischer&Paykel Healthcare (Auckland, New Zealand) RT132 pediatric ventilator tubing. The head model was placed between the artificial lung machine and the ventilator on a pillow at an angle of 30–45° in a half-upward position. Ventilator pre-settings were: NIV-spontaneous/timed (ST) mode in infant modus, inspiratory time 0.8 s at 30 times per min, and varied pressure control and PEEP levels as indicated below. To avoid auto-triggering, the trigger level was set to 5 L/min. The test lung simulator was set at 8 ml/cmH_2_O compliance and 5 cmH_2_O parabolic resistance, within an age-appropriate range for simulation (24). These settings were fixed during all NIV simulation test runs.

### Study Procedure

The two different masks, the anesthetic mask and PerforMax® mask as described earlier were tested in a cross-over design, with the anesthetic mask always tested first to avoid bias introduced by a learning effect. In four separate experiments, three nurses, who are experienced in working with NIV at the PICU of the Amsterdam UMC, and one inexperienced student were asked to participate for placement of the masks on the pediatric head–lung model for NIV as if it were a real clinical setting. The time until satisfactory placement of the masks and initiation of NIV was measured using a stopwatch. During NIV, flow, and pressure waveforms on the screen of the ventilator could be consulted by the nurse to allow adjustment of the position of the mask. In addition, the nurses were allowed to replace the mask in case of evident visible air leaks from the masks, but the nurses were blinded to the air leakage percentages (primary outcome) as measured by the ventilator.

For each mask, NIV was applied at six positive pressure ventilation (pressure control/PEEP) levels (cmH_2_O) for 1-min test runs: 5/5, 10/5, 15/5, 5/10, 10/10, and 15/10. The air leakage percentage was noted twice (at *t* = 0 s and 60 s) for each positive pressure ventilation level. In addition, the user-friendliness of placement of the masks on a 1 to 10 scale as reported by nurses was noted.

### Statistical Analysis

The primary outcome, the percentage of air leakage during NIV simulation, is presented as mean ± SEM. Comparisons between the two masks for each positive pressure ventilation level were performed in GraphPad Prism 9.3.1 by paired *t-*test. A *p* < 0.05 was considered significant.

## Results

### Pediatric Head–Lung Model

**Figure 1:**
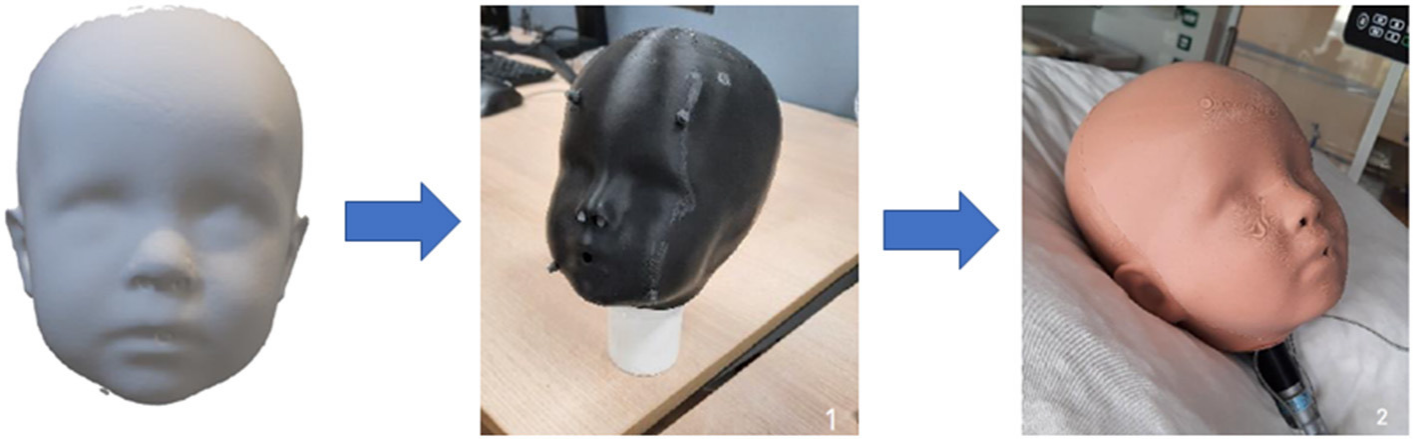
Representation of the production process of the pediatric head model (based on the 3D anthropometric data for an average 1-year-old child) with an outer silicone layer.

**Figure 2:**
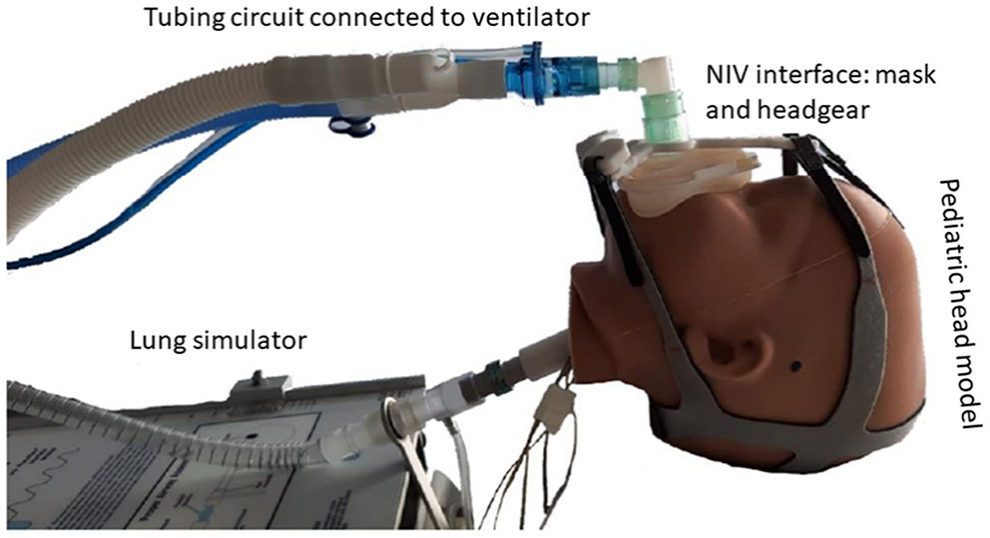
Representation of the pediatric head–lung model is set up with an example of an NIV interface mask connected to mechanical ventilator equipment.

**Figure 3:**
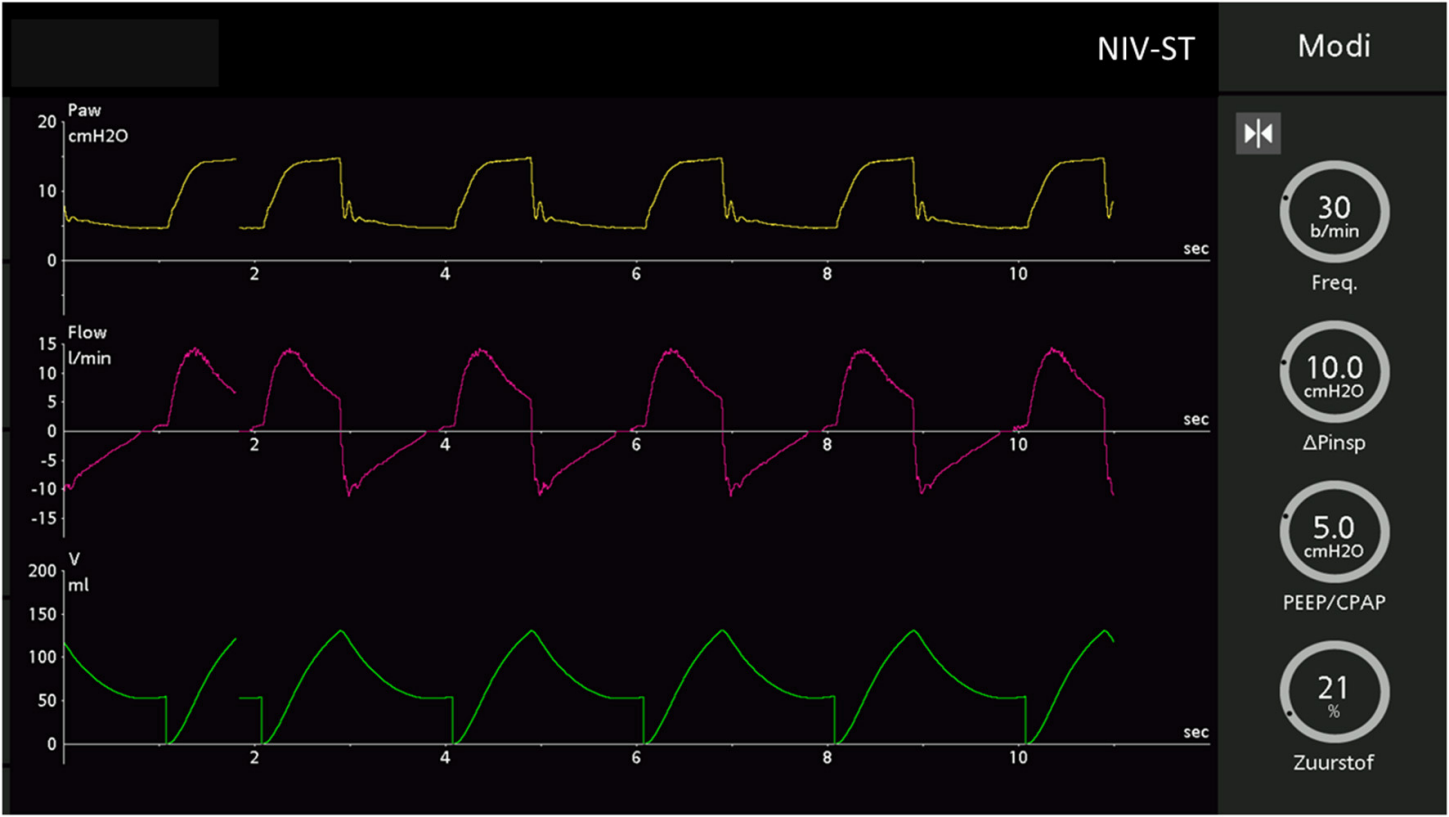
Screenshot from the Hamilton C6 ventilator showing the pressure, flow, and volume waveforms in NIV–ST mode during pediatric NIV simulation using the head–lung model.

The production process from the 3D scan shape model, based on the anthropometric data in the DINED database, to printing the inner head with molded outer silicone layer for the human skin texture is represented in Figure 1. Subsequently, this pediatric head model was connected to the lung simulator, and the ventilator equipment with tubing circuit was connected to any desired NIV interface while placed on the head model (Figure 2). Using this model, we were able to simulate pediatric NIV (Figure 3).

### Air Leak Performance During Simulated NIV

**Figure 4:**
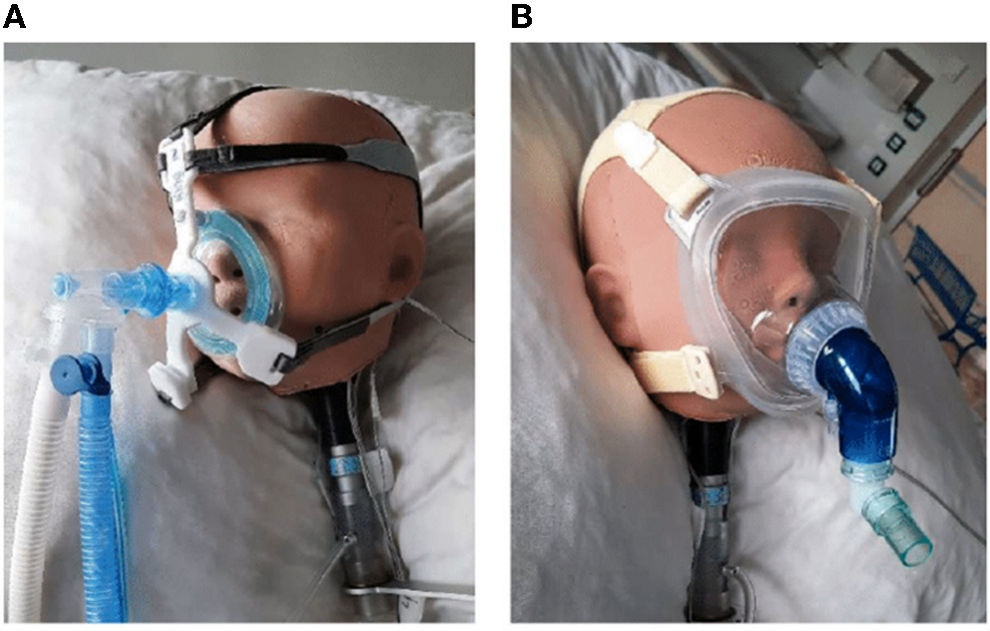
**(A)** The simple anesthetic mask (Ambu®King Mask, size 2) placed on the pediatric head model by a five-point headgear by our in-house 3D-printed quick release adaptor as previously described (22). **(B)** The PerforMax®, size XS, total face mask placed on the pediatric head model.

In this study, we aimed to determine the air leak performance of the simple anesthetic mask with a 3D-printed quick release adaptor connected to a five-point headgear (Figure 4A) as compared to the PerforMax® total face mask (Figure 4B). Both interfaces could be used by the nurses to simulate NIV in the pediatric head–lung model. The mean (±SEM) time to reach a first satisfactory placement on the head model was comparable for both interfaces: 148 s (±23) for the anesthetic mask and 169 s (±44) for the PerforMax® mask. During the NIV simulation, only the PerforMax® mask was adjusted at least once by all nurses. The anesthetic mask scored a 7.6 and the PerforMax® mask scored a 5.3 on a 1-to-10 scoring system for ease-of-use.

**Figure 5:**
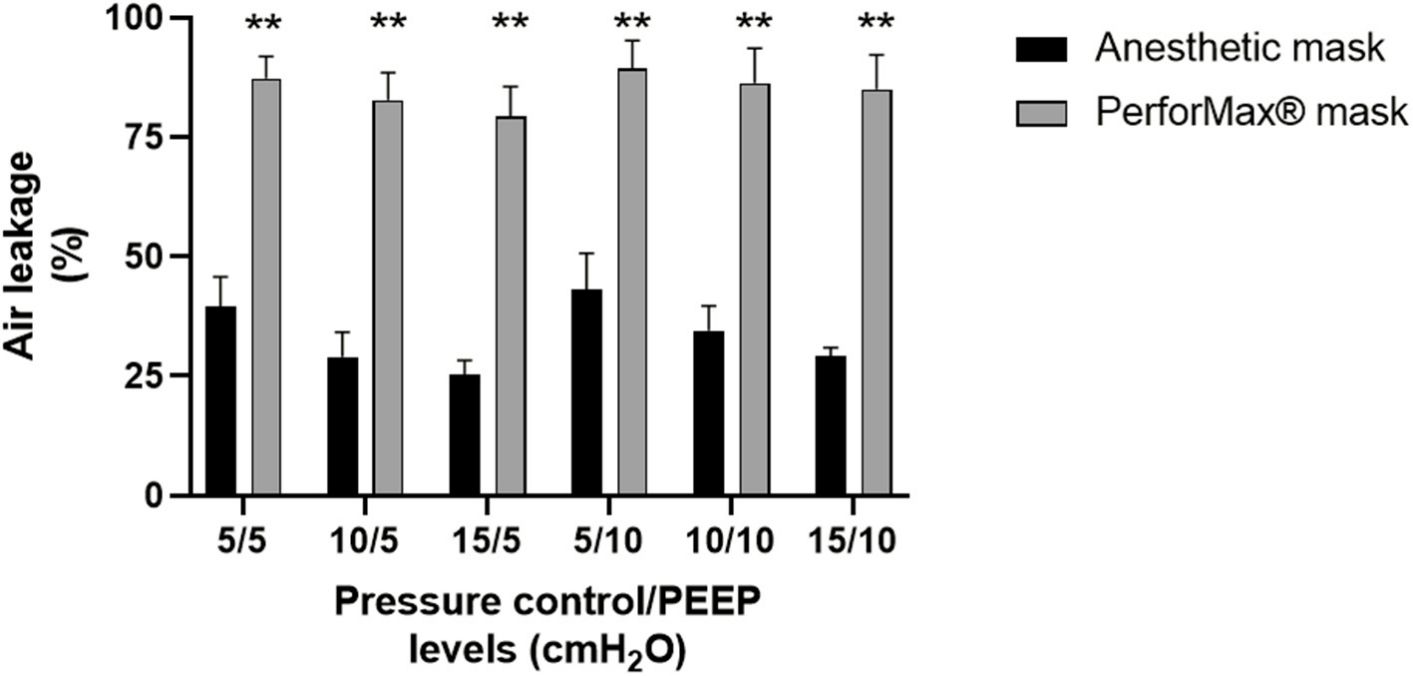
Air leak performance. Mean (SEM) percentages of air leakage measured at various positive pressure ventilation levels (pressure control/PEEP: positive end-expiratory pressure) using the simple anesthetic mask (Ambu®King mask, size 2) with 3D-printed quick release adaptor or the Respironics PerforMax® total face mask size XS during NIV simulation in a head–lung model based on the averaged anthropometric 3D data of a 1-year-old child. ** *p* < 0.01 by paired *t*-test for every positive pressure ventilation level. Data were derived from four separate experiments with four different nurses connecting the two masks in a cross-over design.

The percentage of air leakage as measured by the ventilator during the test runs was significantly lower in the anesthetic mask as compared to the PerforMax® mask at each positive pressure ventilation level. Overall, an absolute reduction of 45–55% in air leakage percentage was observed (Figure 5).

## Discussion

The primary aim of this study was to determine the performance of our recently described simple anesthetic mask with a 3D-printed reusable quick-release adaptor in terms of air leakage, as compared with a commercially available pediatric NIV mask. For this purpose, we used a head–lung model based on the averaged 3D anthropometric data of a 1-year-old child to simulate pediatric NIV. The results further underscore that the simple anesthetic mask may be a reasonable alternative in pediatric NIV in the acute setting. The pediatric head–lung model serves as a promising tool for future investigations into strategies that optimize and personalize NIV interfaces for children.

The choice for an adequate, proper fitting interface is imperative to successfully support patients with NIV (9, 11). Severe air leakage by suboptimal mask sealing is a predictor of NIV failure with the need to escalate to invasive mechanical ventilation (25). Compensation algorithms by modern ventilators are not always able to fully address such leaks (26). Large air leaks affect ventilator volume or pressure targets, lead to patient-ventilator asynchrony, and often result in increased inspiratory flows, which in turn again increase the degree of air leakage (27). Moreover, air leaks may cause dry mucosa of the mouth, nose, and eyes and contribute to patient discomfort. On the other hand, tightening the mask straps to improve sealing can lead to painful pressure skin injury and discomfort as well. This fine balance between minimizing air leakage while avoiding high pressure from the mask is currently one of the major challenges in NIV. In the PICU, this issue is especially apparent in children of young age or with specific facial features as part of a (genetic) syndrome.

As a means to improve NIV treatment success, over the last years, we have initiated a research and development platform focusing on optimizing NIV mask fitting in children with acute respiratory failure. Ultimately, ultrarapid 3D scanning and printing techniques could facilitate full or semi-personalization of these masks, even in the acute phase of critical illness in the PICU. Other groups have explored similar strategies in adult patients and volunteers using stereolithography 3D printing and MRI data (16, 21, 28). By the development of a pediatric head–lung model, as described in this study, we may be able to more readily test several aspects related to the performance of new designs, materials, and full prototypes. In particular, air leakage and ease-of-use, and possibly skin pressure by integrated skin sensors in the future, maybe studied with this model. Head models based on 3D anthropometric data for children of all ages can now be constructed, as well as head models based on specific (abnormal) facial features.

Today, personalized 3D-printed, biocompatible NIV masks are not yet readily available for application in the PICU. As such, inpatient cases where a nasal mask is simply inadequate to provide a sufficient level of respiratory support, we are currently left with commercially available pediatric NIV masks, such as the Respironics PerforMax® total face masks for children elder than 1 year of age. As we frequently observe quite large air leaks from such masks in daily clinical practice, we started to use an alternative interface: a simple, relatively cheap, anesthetic mask connected to a headgear *via* an in-house 3D-printed reusable quick-release adaptor. Recently, we described this interface as part of the successful NIV treatment in a 4-year-old boy with cardiofaciocutaneous syndrome and rhinovirus-associated hypoxic acute respiratory failure (22). Here, we used the pediatric head–lung model to more objectively investigate the extent of air leakage from this simple anesthetic mask during simulated pediatric NIV.

Although the simple anesthetic mask outperformed the Respironics PerforMax® total face mask in terms of air leakage at all positive pressure levels tested, we need to stress the limitations of this simulated setting. First, the pediatric head–lung model cannot simulate movements that may shift the NIV mask and connection to the headgear, thereby decreasing the seal. While critically ill children certainly move less than their healthy counterparts, discomfort, increased work-of-breathing, and airway hygiene procedures (e.g., suctioning) frequently lead to head movements and mask dislocations. This will increase air leakage in clinical practice. Second, the interior of the head model does not fully reflect the upper airway cavities, which will affect flows and volume/pressure targets. As such, care should be exercised in interpreting the absolute respiratory mechanics from NIV simulation with this model (24). Third, we covered the head model with a silicone layer to mimic the flexible skin of a human as much as possible, but subtle differences in the elastic behavior of this artificial layer may affect the mask seal. While being an improvement over a rigid head model, a uniform thickness silicone layer does not fully capture the variation in soft tissue thickness across the face. Future work could investigate using actual bone geometry as the underlying rigid structure in the head model. Nevertheless, all these issues are true for any NIV interface tested, and we, therefore, believe comparisons, in particular on-air leakage, in the performance between different masks and prototypes can be sufficiently made. Fourth, future integration of skin pressures sensors should further enhance the usefulness of this model. Finally, we need to mention the rather high air leakage percentages observed with the Respironics PerforMax® total face mask in this setting. Although this high degree of air leakage is certainly not rare in our clinical experience, this will obviously not be the case in all patients.

In conclusion, by use of a pediatric head–lung model to simulate NIV in a 1-year-old child we showed that our previously described interface comprising a simple anesthetic mask with a 3D-printed reusable quick-release adaptor has lower air leakage at various positive pressure levels, as compared with a commercially available pediatric NIV mask. Both this alternative NIV interface, as well as the pediatric head–lung model deserve further exploration to optimize NIV interface fitting in critically ill children in the future.

## Data Availability Statement

The original contributions presented in the study are included in the article/supplementary material, further inquiries can be directed to the corresponding author/s.

## Author Contributions

RH: study inception, design, coordination, measurements, and writing the first draft of the manuscript. LG: 3D data acquisition and modeling database. TH: DINED Mannequin tool design. MG, RK-B, and DM: study design and NIV simulation set-up. CD: study inception, design, and engineering/3D printing. RB: study inception, design, coordination, and writing the first draft of the manuscript. All authors contributed to reviewing and writing the manuscript.

## Funding

The PICU Amsterdam UMC receives support for a project to personalize NIV mask interfaces in children from the Stichting Steun Emma Kinderziekenhuis (Foundation for support of the Emma Children's Hospital). The TU Delft received support from ClickNL in the frame of the field lab on Ultra Personalized Products and Services (http://upps.nl).

## Conflict of Interest

The authors declare that the research was conducted in the absence of any commercial or financial relationships that could be construed as a potential conflict of interest.

## Publisher's Note

All claims expressed in this article are solely those of the authors and do not necessarily represent those of their affiliated organizations, or those of the publisher, the editors and the reviewers. Any product that may be evaluated in this article, or claim that may be made by its manufacturer, is not guaranteed or endorsed by the publisher.
